# Implementation of Kalman Filtering with Spiking Neural Networks

**DOI:** 10.3390/s22228845

**Published:** 2022-11-16

**Authors:** Alejandro Juárez-Lora, Luis M. García-Sebastián, Victor H. Ponce-Ponce, Elsa Rubio-Espino, Herón Molina-Lozano, Humberto Sossa

**Affiliations:** Instituto Politécnico Nacional, Centro de Investigación en Computación, Mexico City 07738, Mexico

**Keywords:** Kalman filter, artificial intelligence, spiking neural networks, robotics, dynamics

## Abstract

A Kalman filter can be used to fill space–state reconstruction dynamics based on knowledge of a system and partial measurements. However, its performance relies on accurate modeling of the system dynamics and a proper characterization of the uncertainties, which can be hard to obtain in real-life scenarios. In this work, we explore how the values of a Kalman gain matrix can be estimated by using spiking neural networks through a combination of biologically plausible neuron models with spike-time-dependent plasticity learning algorithms. The performance of proposed neural architecture is verified with simulations of some representative nonlinear systems, which show promising results. This approach traces a path for its implementation in neuromorphic analog hardware that can learn and reconstruct partial and changing dynamics of a system without the massive power consumption that is typically needed in a Von Neumann-based computer architecture.

## 1. Introduction

System dynamics can be represented as a set of differential equations in a space–state manner, and they are defined by using several techniques that explore the system’s energetic relationships, such as Newtonian, Lagrangian, or Hamiltonian mechanics. However, trying to describe some phenomena correctly without knowing the governing modeling equations or without a proper selection of the space–state variables results in inaccurate representations or a complex set of equations that could be represented in a more straightforward but unknown form [1]. Data-driven system modeling refers to a set of optimization techniques intended to obtain a system’s description based on data observations and measurements of the system’s evolution. For example, the sparse identification of nonlinear dynamics (SINDY) [2,3] creates a matrix filled with proposed functions and a coefficient matrix, which must be obtained by using well-documented optimization techniques, such as least-square optimization, to replicate the proportionated data as closely as possible.

Artificial neural networks (ANNs) have tackled this challenge on multiple frontiers. Physics-informed neural networks (PINNs) use prior knowledge of the laws of general physics as a regularization agent during their training process, thus limiting the space of admissible solutions [4]. For instance, a Kalman filter (KF) is a model-based technique that allows sensor fusion in order to construct a full space–state recovery based on preliminary knowledge of the system’s model and the nature of perturbation noise, which is useful for unknown perturbances or noisy sensor measurements [5,6]. In [7], the proposal of KalmanNet replaced parts of the equations of the extended Kalman filter (EKF) with an ANN with gated recurrent units (GRUs) to find the proper Kalman gain matrix that would allow a full state recovery.

For example, robotic systems exhibit changing dynamics during their lifespan due to the attrition of joints or their interactions within a changing environment. Therefore, compact and energy-efficient learning platforms are required for any autonomous robotic solution [8]. However, the Von Neumann computer architecture, which is present in all commercially available computing solutions, separates processing and storage into different functional units. Emulating an ANN, a computing strategy that inherently performs storage and processing as closely as possible creates a data bottleneck, as the interactions of neurons and synapses are represented in terms of massive matrix multiplication. The state-of-the-art research on ANNs is performed with sizable graphic processing units (GPUs) or multiple-core computing solutions, the power consumption of which is estimated to surpass humans’ current energy generation capacity if this rate continues [9].

It is necessary to rethink how to perform computing in a move away from the Turing machine, which requires many layers of abstraction, into parallel hardware with distributed memory [10]. Spiking neural networks (SNNs) are considered the third generation of ANNs. These models reflect complex biological and temporal dynamics in order to construct artificial software/hardware counterparts with the same behaviors as those of neurons and synapses [11]. Neuromorphic computing has emerged as a branch in computer science that aims to create computer architectures that resemble the brain’s energy efficiency, learning plasticity, and computing capacity [12]. This has become the inherited hardware platform for SNNs, which usually dictate the design of the building blocks for hardware solutions [13] that are usable for robotic platforms. For instance, in [14,15], an SNN learned the inverse kinematics (IKs) of a robotic arm manipulator, which are usually hard to obtain. While such networks can be used to reconstruct IK values, the extraction of the specific modeling functions from the network is still a research topic.

On this basis, the development of neuromorphic accelerators based on existing complementary metal-oxide semiconductor (CMOS) digital technology is enabling research in neuromorphic computing. such technologies usually include peripheral devices and software/hardware bridges with conventional computing architectures, thus enabling network analysis, performance measurement, and reconfiguration, such as in Intel’s Loihi 2 chip with 130 million silicon neurons and 256 million synapses [16], which is programmable with the Lava neuromorphic compiler, Truenorth from IBM with 1 million neurons and 256 synapses [17], or BrainChip’s Akida [18], which was built with TSCM’s 28 nm technology, among others. These have been used to obtain remarkable results in robotics [19], sensing, and classification tasks. However, as the construction of these accelerators relies on expensive proprietary CMOS chip technologies, they face the same scaling and energy consumption limits [20] as those of their Von Neumann counterparts.

From the perspective of analog electronics, a passive electric device called a memristor [21], which was theorized by Leon Chua, can be used for in-memory computing. It maintains its internal conductance state based on the current that has flowed through its terminals. These passive devices can be used in high-density crossbar arrays (CBAs), which can perform parallel vector–matrix multiplication with ultra-low energy consumption. Analog neurons and synapses have been assembled to compute values that rely on current summation rather than digital Boolean operations [20,22], resulting in some already-built analog neuromorphic architectures [23,24].

This article explores the concept of KalmanNet by entirely replacing its ANN architecture with a proposed SNN architecture to assemble biologically plausible neuron and synapse models. In addition, we propose a new differentiable function for modeling the encoding/decoding algorithms. The proposed architecture was tested in numerical simulations using two well-known nonlinear systems, which showed the feasibility of the solution. At the same time, its possible construction requirements were explored with the aim of its construction in neuromorphic hardware that would be capable of online learning in a space- and energy-efficient neuromorphic hardware solution.

This article is structured as follows: In Section 2 (Materials and Methods), neurons, synapses, and encoding/decoding models are described, and it is shown how these can be interconnected to create the proposed network solution. Section 3 (Results) shows numerical simulations with the nonlinear canonical Van der Pol and Lorenz systems used to test the capabilities of the architecture. Section 4 discusses the results, while Section 5 closes this work by showing our conclusions and proposing future research.

## 2. Materials and Methods

In this section, we start by reviewing how neurons, synapses, and learning rules are modeled. Then, we show encoding/decoding algorithms to determine the current input for the neurons in order to represent the signals used in our proposal. Next, the Kalman filter algorithm is illustrated. After the building blocks are introduced, our proposal is shown at the end of this section.

### 2.1. Neuron Modeling

Leaky Integrate and Fire (LIF) is one of the simplest models available for neuron modeling. It resembles the dynamics of a low-pass filter [25], as it considers a neuron as a switching resistance–capacitance circuit that is governed by:

(1) $$\tau_{m}\frac{dv_{m}\left(t\right)}{dt}=E_{L}-v_{m}\left(t\right)+R_{m}I_{syn}\left(t\right)$$

In (1), $v_{m}\left(t\right)$ represents the membrane’s potential, $E_{L}$ is the membrane’s potential at rest, $\tau_{m}=R_{m}C_{m}$ stands for the membrane’s temporal charging constant, $R_{m}$ is the membrane’s resistance, and $C_{m}$ is the membrane’s capacitance. $I_{syn}\left(t\right)$ acts as an excitatory input current for the neuron, which charges the membrane’s potential $v_{m}\left(t\right)$ until it passes a threshold voltage value $v_{th}$, at which point a spike is emitted. The spike’s voltage, $v_{s}\left(t\right)$, is shaped as follows:

(2) $$v_{s}\left(t\right)=v_{spk}\delta \left(t-t^{f}\right)$$

where $t^{f}$ is the last moment at which a spike was produced, whereas δ(·)∈[0,1] is the Dirac delta function that models the impulse’s decay alongside the synapses, which decay from a maximum value $v_{spk}$ at $t=t^{f}$ to zero at the following post-synaptic rate $\tau_{pstc}$:

(3) $$\delta \left(x\right)=e^{-{\left(\frac{x}{\tau_{pstc}}\right)}^{2}}$$

Once the spike is produced, $v_{m}\left(t\right)$ resets to $E_{L}$. The neuron will not spike again during a refractory period $\tau_{ref}$, as it does not admit an excitatory input current. When $I_{syn}\left(t\right)=0$, $v_{m}\left(t\right)\to E_{L}$.

**Figure 1:**
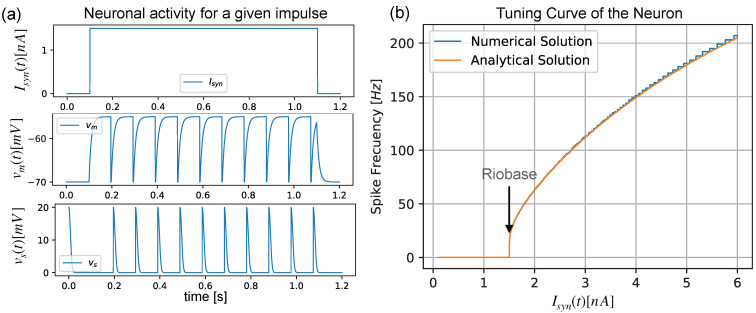
(**a**) Membrane voltage $v_{m}\left(t\right)$ and spike voltage $v_{s}\left(t\right)$ of an LIF neuron for an excitatory input current of $I_{syn}=1.5001nA$. (**b**) Tuning curve of the neuron, which shows the *riobase* value for the parameters given in Table 1.

Given a connection between the *j*-th and *k*-th neuron by a synapse with a certain conductance value $w_{jk}$ (the modeling of which will be reviewed in Section 2.2), the input current for the postsynaptic neuron will be a function of each spike from the presynaptic neuron and its propagation through the corresponding synapse. For *j* presynaptic neurons, the current $I_{syn}\left(t\right)$ for the *k*-th neuron is modeled by the following expression:

(4) $$\tau_{syn}\frac{dI_{syn}}{dt}=-I_{syn}\left(t\right)+C_{syn}\sum w_{jk}\cdot v_{spk}\cdot \delta \left(t-t_{pre}^{f}\right)$$

where $t_{pre}^{f}$ is the firing time of each presynaptic neuron. Equations (1) and (4) make up the conductance-based LIF model [26], where $\tau_{syn}$ is the injection current time decay and $C_{syn}$ stands for the temporal injection current constant, which models the scale of the current injection of the presynaptic impulses. Figure 1 shows a step impulse of 1.5nA fed to a single neuron, which is modeled by Equation (1), showing its internal state $v_{m}\left(t\right)$ and the produced spike voltage $v_{s}\left(t\right)$. The parameters used for the neuron that is used are provided in Table 1.

#### Frequency Response of the Neuron

To compute how much current has to be fed into the neuron to obtain a given frequency response, first, we need to compute how much time it will take for the neuron to pass from a resting stage to a firing stage by analytically solving the differential Equation (1):

(5) $$v_{m}\left(t\right)=E_{L}+R_{m}I_{syn}+C_{1}e^{-t/\tau_{m}}$$

For t=0, we can rewrite $C_{1}=v_{m}\left(0\right)-E_{L}-R_{m}I_{syn}\left(t\right)$. Setting the initial conditions to the values of $v_{m}\left(0\right)=E_{L}$, and $v_{m}\left(t\right)=v_{th}$ in Equation (5), we can solve for *t* to obtain the expression of the membrane’s potential charging time $t_{spk}$:

(6) $$t_{spk}=-\tau_{m}\ln\left(\frac{v_{th}-E_{L}-R_{m}I_{syn}\left(t\right)}{-R_{m}I_{syn}\left(t\right)}\right)$$

As the firing frequency $f_{spk}=1/T_{spk}$, where $T_{spk}=\tau_{ref}+t_{spk}$, we have:

(7) $$f_{spk}\left(t\right)=\frac{1}{\tau_{ref}-\tau_{m}\ln\left(\frac{v_{th}-E_{L}-R_{m}I_{syn}\left(t\right)}{-R_{m}I_{syn}\left(t\right)}\right)}$$

Equation (7) computes the frequency response of a neuron given a certain current. The inverse function computes the opposite—the amount of current needed for a given frequency:

(8) $$I_{syn}\left(t\right)=\frac{v_{th}-E_{L}}{R_{m}\left(1-e^{\frac{\tau_{ref}-\frac{1}{f_{spk}\left(t\right)}}{\tau_{m}}}\right)}$$

Figure 1b shows the firing response of a neuron with respect to the firing frequency response for a given excitatory input current; this is called a *tuning curve*, and it was obtained using Equation (7) (analytical solution) and a numerical simulation of Equation (1), with a sweep from 0 A to 6 nA, using the neuron parameter values that appeared in Table 1. Setting f=1 Hz, we obtain $I_{r}=1.5$ nA. This is called the *riobase* current of the neuron.

### 2.2. Synapse Modeling

Spike-time-dependent plasticity (STDP) is a Hebbian learning algorithm that reflects how a synapse’s conductivity increases or decreases according to the neuron spiking activity [27]. Given the *j*-th layer of *N* presynaptic neurons and the *k*-th layer of *M* postsynaptic neurons, a matrix of $W=\left[w_{jk}\right]\in R^{N\times M}$ synapses will form between them, and its weight value will be modified by:

(9) $$\Delta w\left(\Delta t\right)=\left\{\begin{array}{c} A_{+}e^{-\frac{-\Delta t}{\tau_{+}}},\forall \Delta t\geq 0\\ A_{-}e^{\frac{\Delta t}{\tau_{-}}},\forall \Delta t<0 \end{array}\right.$$

(10) $$w_{ij}=\sum_{t_{pre}^{f}}\sum_{t_{post}^{f}}\Delta w$$

In Equation (9), $\Delta t=t_{post}^{f}-t_{pre}^{f}$ is the difference between the firing times of the postsynaptic and presynaptic neurons. $\tau_{+},\tau_{-}$ are the *long-term potentiation (LTP)* and *long-term depreciation (LTD)* constants, which map the decay effect of a spike in the modification of the weight. For each spike, the synaptic weight is then modified by a learning rate of $A_{+},A_{-}$. When $A_{+}=A_{-}$ and $\tau_{+}=\tau_{-}$, the response is symmetrical, that is, the synapse modifies its value equally for presynaptic or postsynaptic spikes. STDP is included in the unsupervised learning paradigm [8], as there is no *teaching signal* involved, rather than the input and output signals to be processed.

### 2.3. Reward-Modulated STDP (RSTDP)

In order to introduce a teaching signal, some modifications to the STDP algorithm were described in [8] based on dopamine’s modulation of the learning ability in the synapses observed in biological systems. Starting from Equation (9), an eligibility trace *E* can be defined by taking into account only the last pre- and postsynaptic spike potentials at time *t*:

(11) $$\frac{dE}{dt}=-\frac{E(t)}{\tau_{E}}+A_{+}v_{spk}\delta \left(t-t^{pre}\right)+A_{-}v_{spk}\delta \left(t-t^{post}\right)$$

The eligibility trace is intended to model the tendency of the change in the synaptic weight value as a transient memory of all of the spiking activity, where $\tau_{E}$ depicts its decay time. The rate of change in the synaptic weights *w* is then obtained as follows:

(12) $$\frac{dw}{dt}=R\left(t\right)\times E\left(t\right)$$

where R(t)∈[−1,1] is a reward signal, which is defined according to the network’s objectives. It is worth mentioning that when R=0, learning is deactivated, as no change in synapses is produced. When R=−1, the weights are forced to converge in the opposite direction. Finally, when R=1, the eligibility trace remains unaltered.

**Figure 2:**
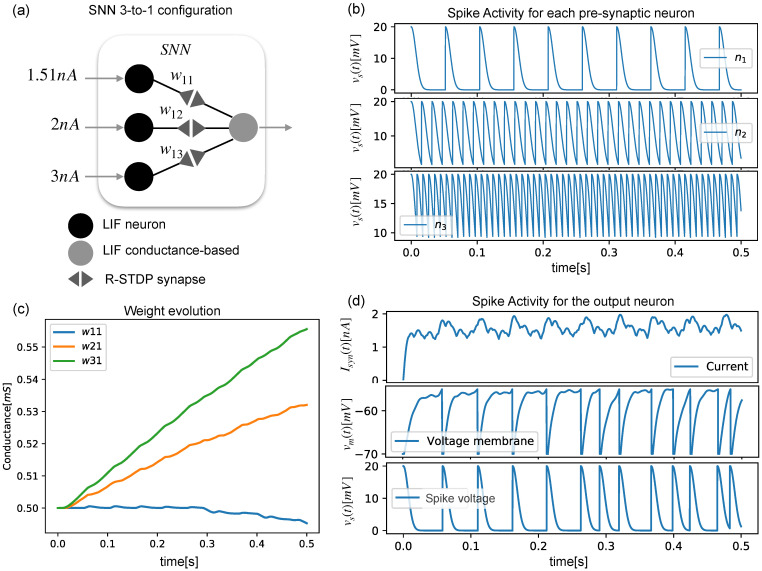
(**a**) An SNN with three LIF neurons in the input layer and one output layer. (**b**) Spiking activity of the first layer. (**c**) Evolution of the weight of the synapse. (**d**) Neural activity (input current, membrane voltage, and spike voltage) of the output neuron.

Three presynaptic neurons and one postsynaptic neuron were arranged as shown in Figure 2a, and they produced different spiking activities (Figure 2b), showing how the output neuron’s membrane voltage accumulated with each arriving spike (Figure 2d). As each neuron spiked with a different frequency, the synaptic weight evolved into different values (Figure 2c).

### 2.4. Encoding and Decoding in Spiking Neural Networks

Given an analog input signal that is intended to be processed by an SNN, a proper truly excitatory input current that represents every possible value from the input signal should be computed (encoding). Furthermore, the spiking activity of a neuron must be interpreted back from the spiking domain into the analog domain in order to interact with external systems (decoding).

#### Encoding Algorithm

There are several encoding and decoding algorithms that have been proposed in the literature. Some of them have the intention of reflecting biological plausibility, or easing the construction of neuromorphic devices. *Rate-based* encoding takes an input signal $x\left(t\right)\in \left[x_{min},x_{max}\right]$ and a minimum and maximum spiking frequency operation of the neuron $F=[F_{min},F_{max}]$, and it uses Equations (7) and (8) to encode/decode, respectively. Nonetheless, the encoding process can be performed as a function of the variability of the signal, which can be divided into *phase encoding and time-to-first-spike* encoding, among others [27,28]. Step-forward encoding, which was described in [29], is a temporal encoding algorithm that harnesses the low-pass filter dynamics of the LIF neuron in conjunction with a temporal encoding methodology. The input signal x(t) is compared with an initial baseline signal $x_{b}\left(t\right)$ and a sensibility encoding threshold value $x_{th}$. If $x\left(t\right)>x_{b}+x_{th}$, a certain current $I_{syn}^{+}$ is fed into an LIF neuron, which is denoted as $N^{+}$. However, if $x\left(t\right)<x_{b}-x_{th}$, a fixed current $I_{syn}^{-}$ is then fed into another LIF neuron (denoted as $N^{-}$). Therefore, $N^{+}$ will only spike for a growing signal, while $N^{-}$ will spike for decreasing signals. In this work, the conditional part of this encoding algorithm is replaced with differentiable functions with the aim of easing future mathematical convergence analyses. Setting $\alpha =\tanh(c\cdot \left(x\left(t\right)-x_{b}\left(t\right)\right)$,

(13) $$I_{syn}^{+}\left(t\right)=I_{r}\left(1+\alpha \right)$$

(14) $$I_{syn}^{-}=I_{r}\left(1-\alpha \right)$$

where *c* is a slope modulation constant, which, for high values, approximates tanh(·) function as closely as the hardlim function. The baseline signal for the encoding is then updated by:

(15) $$x_{b}\left(t\right)=x_{b}\left(t-1\right)+\alpha x_{th}$$

**Figure 3:**
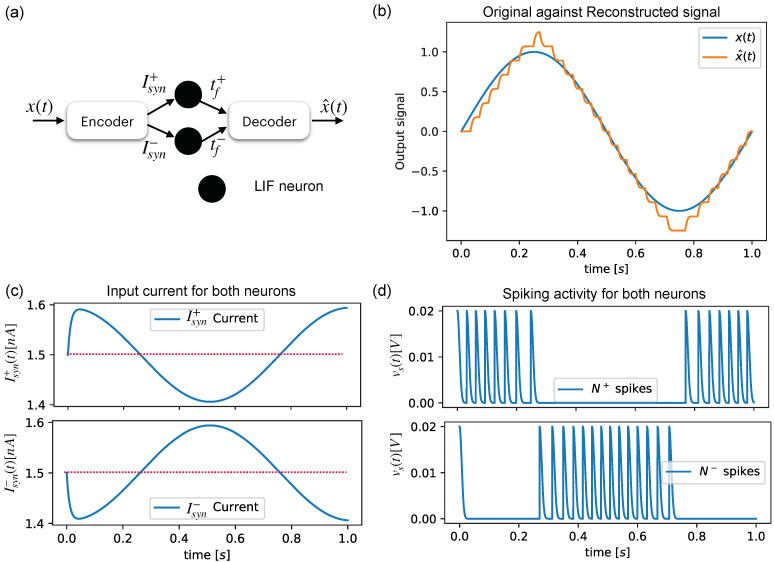
Signal reconstruction using neurons and encoding/decoding algorithms. (**a**) Assembly of the encoding/decoding, which alternate the input currents of two different neurons. (**b**) Comparison between the original signal x(t) and reconstructed signal $\hat{x}\left(t\right)$. (**c**) Spiking activity response for each neuron. (**d**) Input currents $I_{syn}^{+},I_{syn}^{-}$ for the neurons (Blue) versus the riobase (red dotted). (**d**) Output spikes for each neuron in the assembly.

For decoding, the output signal $\hat{x}\left(t\right)$ is computed with the following expression:

(16) $$\hat{x}\left(t\right)=\hat{x}\left(t-1\right)+x_{th}\delta \left(t-t_{f}^{+}\right)-x_{th}\delta \left(t-t_{f}^{-}\right)$$

where $t_{f}^{+}$ stands for the spiking time of the $N^{+}$ neuron and $t_{f}^{-}$ is the firing time of the $N^{-}$ neuron. Figure 3a shows a simple configuration for reconstructing an input sine signal, which is shown in Figure 3b, by using the spiking activities of two neurons (Figure 3c) that are fed by an encoding block composed of Equations (13) and (14), which feed $N^{+}$ and $N^{-}$ with the current levels shown in Figure 3d.

### 2.5. Discrete Extended Kalman Filter

The discrete *extended Kalman filter* (EKF) allows full state estimation of system dynamics based on partial and/or noisy measurements. Given a system represented in a discrete space–state manner [5],

(17) $$x_{k}=f\left(x_{k-1},u_{k}\right)+w_{k}$$

(18) $$y_{k}=h\left(x_{k}\right)+v_{k}$$

where $x_{k}\in R^{n}$ is the state vector of the system, and f(·) is nonlinear and describes the evolution of the dynamics given the state value at the previous timestep $x_{k-1}$ and a control input $u_{k}\in R^{n}$. $y_{k}\in R^{m}$ is the available output of the system, which is described by h(·). w∼N(0,Q) and v∼N(0,R) are additive white Gaussian noise (AWGN) with a covariance matrix $Q\in R^{n\times n}$ and $R\in R^{m\times m}$, respectively, representing the system uncertainties given by perturbations or noisy measurements. The EKF algorithm retrieves an estimation ${\hat{x}}_{k|k}$ that ideally tends to ${\hat{x}}_{k|k}\to x_{k}$. As f(·),h(·) are nonlinear, the EKF uses a linearized version of the system’s model by obtaining their respective Jacobians:

(19) $$A=\frac{\partial f}{\partial x}{|}_{{\hat{x}}_{k-1|k-1},u_{k}}$$

(20) $$C=\frac{\partial h}{\partial x}{|}_{{\hat{x}}_{k|k-1}}$$

where ${\hat{x}}_{k-1|k-1}$ is the estimation of the EKF in the previous timestep. The *discrete EKF* is a two-step procedure involving a *prediction* and an *update*:

1. *Prediction*: First, a preliminary estimation ${\hat{x}}_{k|k-1}$, ${\hat{y}}_{k|k-1}$ is computed by:
  (21) $${\hat{x}}_{k|k-1}=f\left({\hat{x}}_{k-1|k-1},u_{k}\right)$$
  (22) $${\hat{y}}_{k|k-1}=h\left({\hat{x}}_{k|k-1}\right)$$
  Then, a covariance estimate $P_{k|k-1},S_{k|k-1}$ is computed, and the noise covariance matrices Q,R and the estimate in the previous timestep $P_{k-1|k-1}$ are taken into account:
  (23) $$P_{k|k-1}=A\cdot P_{k-1|k-1}\cdot A^{T}+Q$$
  (24) $$S_{k|k-1}=C\cdot P_{k|k-1}\cdot C^{T}+R$$
2. *Update*: The second step consists of computing the Kalman gain matrix $\kappa \in R^{n\times m}$ with
  (25) $$\kappa =P_{k|k-1}\cdot C^{T}\cdot S_{k|k-1}^{-1}$$
  in which the difference between the measurable output and estimated output of the prediction step is used:
  (26) $$\Delta y_{k}=y_{k}-{\hat{y}}_{k|k-1}.$$
  We can obtain a final estimation ${\hat{x}}_{k|k}$ that considers errors in measurement and noise statistics:
  (27) $${\hat{x}}_{k|k}={\hat{x}}_{k|k-1}+\kappa \cdot \Delta y_{t}$$
  Finally, the moment of the prediction $P_{k|k}$, which will be used for the next timestep in *prediction*, is computed:
  (28) $$P_{k|k}=P_{k|k-1}-\kappa \cdot S_{k|k-1}\cdot \kappa^{T}$$

In order to successfully reconstruct the full state, both the KF and EKF demand full knowledge of the system dynamics, as the correct characterization of perturbations and measurement noise can become cumbersome or unavailable in real scenarios.

### 2.6. Proposed Kalman-Filtering SNN Structure

**Figure 4:**
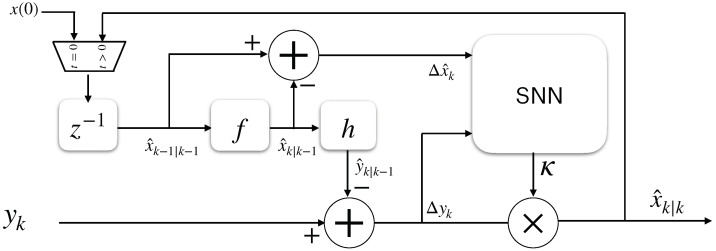
Block diagram of the Kalman filter in which the typical Kalman gain-obtaining procedure is replaced by an SNN.

An SNN structure of an EKF that replaces Equations (23), (24), and (28) is shown in Figure 4. First, the error between the current and prior estimations is defined for all of the space–state variables:

(29) $$\Delta {\hat{x}}_{k|k}={\hat{x}}_{k|k}-{\hat{x}}_{k|k-1}$$

Then, $\Delta \hat{x}$ and $\Delta y_{t}$ are stacked into an input vector $s_{in}$ for the SNN as follows:

(30) $$s_{in}=\left[\Delta {\hat{x}}_{1},...,\Delta {\hat{x}}_{n},\Delta y_{1},...,\Delta y_{m}\right]$$

**Figure 5:**
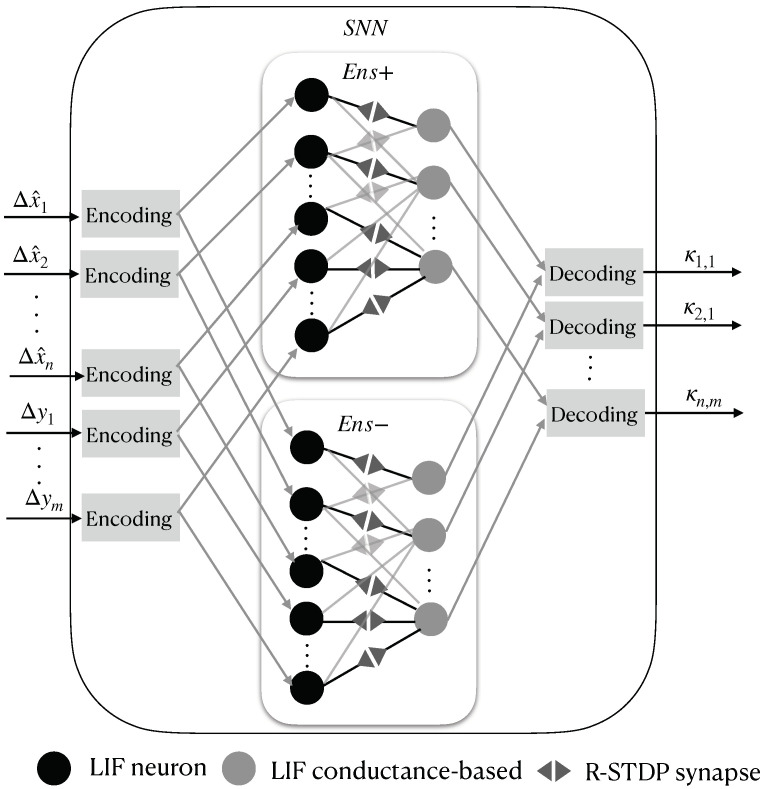
Proposed SNN network architecture for finding the values of the Kalman gain matrix.

This vector is encoded using Equation (13), which produces excitatory input current vectors for two ensembles of neurons inside the SNN, which are called Ens+ and Ens−, and they spike for increasing and decreasing input signals, respectively. Both ensembles count with two densely connected LIF neuron layers—the *j*-th layer with n+m neurons, which is modeled by Equation (1), and the *k*-th layer with n×m LIF neurons, which is modeled by Equations (1) and (4). These are connected by *RSTDP* synapses, as depicted in Equations (11) and (12), with the reward signal set to R(t)=1. The spikes of the *k*-th layer from Ens+ and Ens− are finally decoded with Equation (16) to obtain each value of the Kalman gain matrix in order to properly reconstruct the full state vector of the system. Figure 5 shows the described SNN structure.

## 3. Results

In order to show the performance of the proposal, two nonlinear systems were used. For each system, the nonlinear equations were simulated to create noiseless ground-truth data x(t). Then, the resulting vector was noised as described in (17) and (18) by setting $w_{k},v_{k}$ with the diagonal covariance matrices Q,R as follows:

(31) $$Q=I\cdot q^{2},\;\;\;\;\;R=I\cdot r^{2},\;\;\;\;\;\nu =\frac{q^{2}}{r^{2}}$$

where ν=1 would imply that the state noise and the observation noise have the same variance, i.e., $q^{2}=r^{2}$. The resulting contaminated data then corresponded to a system with noisy measurements and unknown perturbations. The simulation was intended to compare the performance of a standard EKF against the SNN proposal under equal conditions; that is, only noisy measurements were provided. The SNN had to be able to recover this information, while for the EKF, Q,R were set as identity matrices, as these were supposed to be unknown.

To create the system’s synthetic data, as the used models were shaped with $\dot{x}=A\left(x,u\right)\cdot x$, the solution of the nonlinear system could be expressed as a Taylor series expansion with five terms, as in [7], assuming that for a small timestep Δt, f(x(t))≈f(x(t+Δt)). By doing this, we obtained a system that shaped as described in Equation (17).

For the SNN, the neuron parameters in Table 1 were used. The synapse, encoding/decoding, and simulation parameters are found in Table 2. The synapses were randomly initialized in the range of $\left[w_{min},w_{max}\right]$. To display the neural activity, the observed spike frequency for each neuron $f_{obs}$ was computed as follows:

(32) $$f_{obs}=\left(n_{T}\right)\left(\frac{1}{T_{obs}}\right)$$

where $n_{obs}$ counts how many spikes were produced inside a period of length $T_{obs}=50$ ms. The procedure was repeated for the whole simulation timeline of t=60 s, with simulation a timestep of $\Delta t=1\times 10^{-4}$ s.

The simulation scripts were coded from scratch using Python (v+3.8) [30] and the Numpy (v+1.20) and Sympy (v+1.8) [31] libraries. However, during our testing, the Lorenz system’s SNN network was also coded using the SNNtorch (v+0.5.3) library [32]. The resulting code is available in the Data Availability Statement section.

### 3.1. Van der Pol Simulation

Proposed by electrical engineer and physicist Balthasar Van der Pol, this nonlinear model is used to find oscillations on electric circuits using vacuum tubes, and it can be written in the $\dot{x}=Ax$ form as follows:

(33) $$\left(\begin{array}{c} {\dot{x}}_{1}\\ {\dot{x}}_{2} \end{array}\right)=\left(\begin{array}{ccc} \mu \left(1-\frac{1}{3}x_{1}^{2}\right) & \; & -\mu \\ \frac{1}{\mu } & \; & 0 \end{array}\right)\left(\begin{array}{c} x_{1}\\ x_{2} \end{array}\right)$$

where μ=3 refers to the damping strength of the oscillations. For this test, we set our output to y=[1,0]x, that is, only $x_{1}$ was available for the measurement, while $x_{2}$ was set to be recovered from the system.

**Figure 6:**
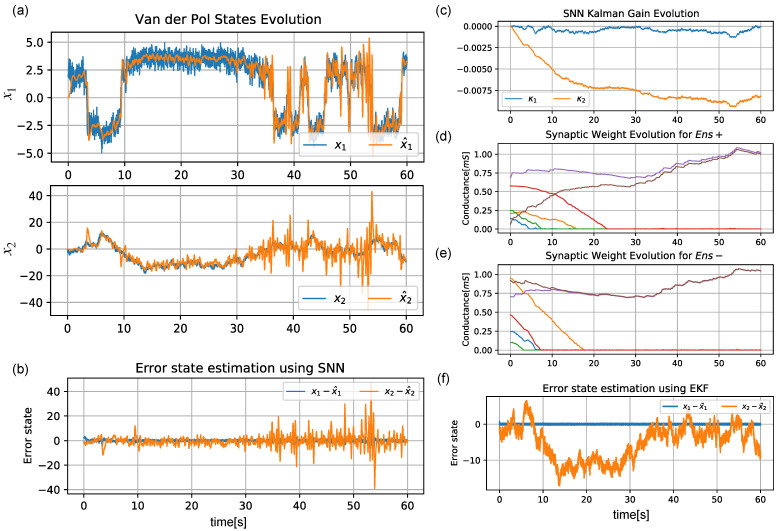
Time evolution of the reconstruction of the Van der Pol oscillator using the proposed architecture in comparison with the ground truth. (**a**) Comparison of the ground truth *x* (blue) with the of the reconstruction $\hat{x}$ (orange) made using the proposed architecture. (**b**) Time error reconstruction $x-\hat{x}$ of the two states of the system. (**c**) Time evolution of each value of the resulting Kalman gain matrix. (**d**,**e**) Weight value evolution over time of the 3×2 synapse set (multiple colors) for Ens+ and Ens−, respectively. (**f**) Time error state estimation of the Van der Pol system using the standard discrete EKF algorithm without knowledge of the covariance matrices Q,R.

Figure 6a shows a correct estimation of $x_{2}$. This can also be seen in the difference $x-\hat{x}$ shown in Figure 6b. The $\kappa \in R^{2\times 1}$ matrix values estimated by the SNN are displayed in Figure 6c; these were obtained by using the spikes of the output layer. The evolution of the synaptic weight is also shown for both ensembles (Ens+, Ens−) in Figure 6d,e, respectively. While the SNN’s estimation became noisier as the time moved forward, it can be seen in Figure 6f that the EKF was not able to properly reconstruct the missing states at any point.

### 3.2. Lorenz System Simulation

A typical dynamic system for testing the obtention of unknown or partial dynamics is the Lorenz attractor, which is composed of the following nonlinear dynamics:

(34) $$\dot{x}=\left(\begin{array}{ccccc} -10 & \; & 10 & \; & 0\\ 28 & \; & -1 & \; & -x_{1}\\ 0 & \; & x_{1} & \; & -\frac{8}{3} \end{array}\right)\left(\begin{array}{c} x_{1}\\ x_{2}\\ x_{3} \end{array}\right)$$

For this system, the EKF can be implemented by using five Taylor series approximation terms, as in [7]. In this test, we set the output to y=[1,0,0]x, which meant that only the $x_{1}$ state was available for measurement. Therefore, $x_{2},x_{3}$ should be recovered.

**Figure 7:**
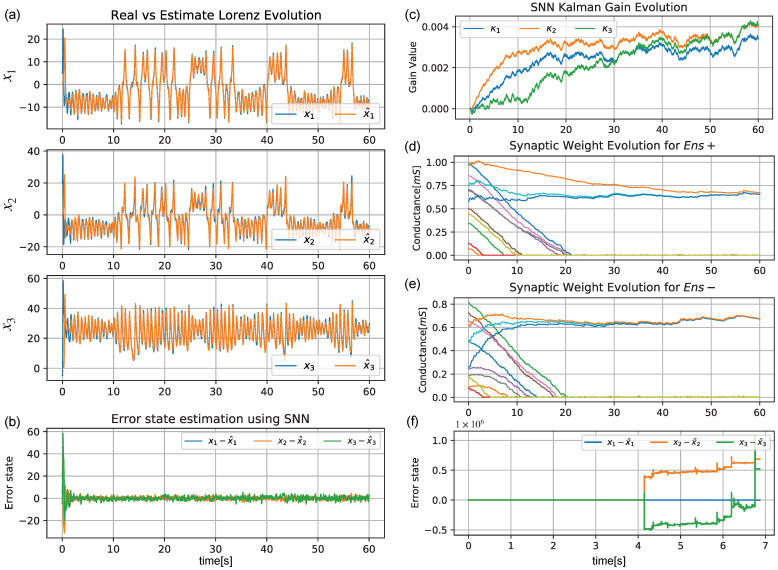
Time evolution of the Lorenz system’s reconstruction when using the proposed architecture in comparison with the ground truth. (**a**) Comparison of the ground truth *x* (blue) with the reconstruction $\hat{x}$ (orange) made by using the proposed SNN architecture. (**b**) Time error reconstruction $x-\hat{x}$ of the three states of the Lorenz system. (**c**) Evolution of each value of the Kalman gain matrix. (**d**,**e**) Weight value evolution over time of the 4×3 synapse set (multiple colors) for Ens+ and Ens−, respectively. (**f**) Time estimation of the Lorenz system using the standard discrete EKF algorithm without knowledge of the covariance matrices Q,R.

Figure 7a shows the estimation of $x_{2},x_{3}$. The error $x-\hat{x}$ is shown in Figure 7b for the three states. The $\kappa \in R^{3\times 1}$ matrix values estimated by the SNN are displayed in Figure 7c. The weight evolution is also shown for both ensembles (Ens+, Ens−) in Figure 7d,e, respectively. In this test, while the error estimation converged to close to zero for the three states (Figure 7b), Figure 7f shows that the EKF quickly diverged to infinity at t=6.9 s due to the missing noise characterization of the system.

## 4. Discussion

A proper full state reconstruction of the space state was achieved. However, some considerations should be addressed. On the one hand, in the KalmanNet structure, the intention being the usage of GRUs is to use them as storage for the internal ANN’s memory *in order to jointly track the underlying second-order statistical moments required for implicitly computing the KG* [7]. In our SNN proposal, the intention is to replace them with the eligibility traces defined by the RSTDP weight update mechanism (Equation (11)), as *E collects the weight changes proposed by STDP*; thus, they represent the potentiation/degradation tendency of the synaptic weight [8].

The energy consumption of an SNN relies on the spiking activity. Therefore, only the necessary spikes should be performed to represent our signals. Rate-based encoding mechanisms return a constant excitatory input current for a constant input signal (no matter its magnitude), resulting in spiking activity for non-changing signals. In temporal encoding schemes, such as the one used in this work, the neurons are only excited based on the rate of change in the input signal. The introduction of Equations (13) and (14) is intended to restrain the excitatory input current of the neurons to minimum and maximum values. In the range of tanh(·)∈[−1,1], for high rates of change, the maximum input current is $I_{syn}=2I_{r}$; according to Equation (7) and the neuron parameters in Table 1, this would correspond to a spike frequency of f≈120 Hz for a maximum input current of $I_{syn}=2\left(1.5nA\right)=3nA$. This can be seen in the resulting spike frequency graphs for both the Van der Pol and Lorenz tests (see the Data Availability section).

However, while the neuron parameters were selected to resemble biological plausibility, proper selection of the encoding/decoding sensibility and the values of the learning rates is fundamental. $x_{th}$ should proportionate enough to $I_{syn}$ to produce a suitable spiking activity, though selecting sufficiently high $A^{+},A_{-}$ values should appropriately modify the synaptic weights with the supplied spikes. Low learning rates may require a higher spike frequency but a higher precision, leading to slow convergence. In contrast, high learning rate values require less spiking activity but lead to a lower precision, which may result in divergence. In addition, to translate this SNN structure into a hardware implementation, the min/max synaptic weight values might be restricted to the observed values in available memristive devices.

A mathematical convergence analysis would determine the boundary conditions for selecting proper parameters. However, the LIF reset condition makes this dynamic non-differentiable, which disables this analysis or the adaptation of back-propagation for SNNs. A way to deal with this is to move the analysis to the frequency domain by solving the LIF model and obtaining the tuning curve produced by Equation (7) and its corresponding graph (Figure 1b). It can be seen that the function only is differentiable in the range of $\left[I_{r},\infty \right)$. In [15], the authors used a polynomial differentiable tuning curve (which can be obtained through least square regression) to avoid this restriction. In this work, the introduction of bounded and differentiable encoding/decoding functions and the usage of two ($Ens^{+},Ens^{-}$) neuron ensembles allowed the usage of this approximation to be avoided, as the dynamics of $Ens^{+}$ are only affected by the growth of input signals, while for $Ens^{-}$, only the decay is processed, thus creating a switching dynamical system [33] that might allow us to propose a Lyapunov candidate function whose derivative is negatively defined.

## 5. Conclusions and Future Work

An SNN-embedded architecture inside the extended Kalman filter algorithm was used to perform the full state recovery of a nonlinear dynamic system based on partial knowledge while assuming unknown but bounded perturbations. Numerical simulations showed the feasibility of the system. While in other works, the encoding/decoding process was performed by using a function approximation relating the input current with the spike frequency, the proposed modifications allow this to be avoided by setting a switched current designation that lets each ensemble of neurons and their respective synapses evolve towards the growth/decay of the SNN input signals while bounding the excitatory input current, thus limiting the spike frequency.

In order to move towards a hardware construction, neuron design with a very large scale of integration (VLSI), the replacement of synapses with memristive devices, and a VLSI design of the encoding/decoding modules would define the building blocks for a system-on-a-chip proposal. However, moving to a hardware implementation in currently available technologies might lead to modifications, such as changes in the values of the memristive range or achievable spike frequencies. Therefore, a framework for mathematical convergence analysis should be defined to study the SNN’s performance with these new parameters. Nonetheless, it was shown that a few resources (in terms of the number of neurons, synapses, and energy consumption) were able to achieve proper performance by taking advantage of existing explainable PINN architectures.

## Figures and Tables

**Table 1:** Neuron, synapse, and encoding parameters.

LIF Model	Parameter Value
Membrane charging constant	$\tau_{m}=10$ ms
Membrane resistance	$R_{m}=10$ MΩ
Capacitance of the neuron	$C_{m}=1$ nF
Threshold voltage of the neuron	$v_{th}=-55$ mV
Resting potential of the neuron	$E_{L}=-70$ mV
Reset potential of the neuron	$v_{reset}=-70$ mV
Spike amplitude	$v_{spk}=\;20$ mV
Postsynaptic current decay time	$\tau_{pstc}=10$ ms
Refractory Period	$\tau_{ref}=2$ ms
**Conductance-Based LIF**	
Time decay of the injection current	$\tau_{syn}=10$ ms
Temporal injection current constant	$C_{syn}=1\times 10^{-5}$

**Table 2:** Encoding/decoding and RSTDP parameters in the simulation.

RSTDP Synapse Model	
Long-term potentiation constant	$A_{+}=1$μS/mSeg
Long-term depreciation constant	$A_{-}=-1$μS/mSeg
Transient memory decay time	$\tau_{E}-=10$ ms
Max. conductance Value	$w_{max}=1$ ms
Min. conductance Value	$w_{min}=1$μs
**SF Encoding and Decoding**	
Encoding sensibility threshold value in a Van der Pol test	$x_{th}=1\times 10^{-4}$
Encoding sensibility threshold value in a Lorenz test	$x_{th}=1\times 10^{-5}$
Decoding sensibility threshold value in both tests	$x_{th}=1\times 10^{-5}$
Slope modulation constant	c=1
**Noise Parameters**	
Measurement noise’s standard deviation	r=0.1
System uncertainties’ standard deviation	q=0.0316

## Data Availability

All of the scripts used in this article are available on the following Github page: https://github.com/LuisGarcia-S/SNN-Kalman-Filtering (accessed on 14 November 2022).

## Abbreviations

The following abbreviations are used in this manuscript:

ANN	Artificial Neural Network
AWGN	Additive White Gaussian Noise
CBA	Crossbar Array
CMOS	Complement Metal-Oxide Semiconductor
EKF	Extended Kalman Filter
GPU	Graphic Processing Unit
GRU	Gated Recurrent Unit
IBM	International Business Machines
IK	Inverse Kinematics
KF	Kalman Filter
LIF	Leaky Integrate and Fire
LTD	Long-Term Depreciation
LTP	Long-Term Potentiation
PINN	Physics-Informed Neural Networks
RSTDP	Reward-Modulated STDP
SINDY	Sparse Identification of Nonlinear Dynamics
SNN	Spiking Neural Network
STDP	Synaptic Time-Dependent Plasticity
TSMC	Taiwan Semiconductor Manufacturing Company
VLSI	Very Large Scale of Integration

## References

[1] Brunton S.L., Kutz J.N. (2022). Data-Driven Science and Engineering: Machine Learning, Dynamical Systems, and Control.

[2] Kaiser E., Kutz J.N., Brunton S.L. (2018). Sparse identification of nonlinear dynamics for model predictive control in the low-data limit. Proc. R. Soc. A Math. Phys. Eng. Sci..

[3] Kaheman K., Kutz J.N., Brunton S.L. (2020). SINDy-PI: A robust algorithm for parallel implicit sparse identification of nonlinear dynamics. Proc. R. Soc. A Math. Phys. Eng. Sci..

[4] Teng Q., Zhang L. (2019). Data driven nonlinear dynamical systems identification using multi-step CLDNN. AIP Adv..

[5] Kálmán R.E., Bucy R.S. (1961). New Results in Linear Filtering and Prediction Theory. J. Basic Eng..

[6] Haykin S. (2001). Kalman Filtering and Neural Networks.

[7] Revach G., Shlezinger N., Ni X., Escoriza A.L., van Sloun R.J.G., Eldar Y.C. (2022). KalmanNet: Neural Network Aided Kalman Filtering for Partially Known Dynamics. IEEE Trans. Signal Process..

[8] Bing Z., Jiang Z., Cheng L., Cai C., Huang K., Knoll A. End to End Learning of a Multi-Layered Snn Based on R-Stdp for a Target Tracking Snake-Like Robot. Proceedings of the 2019 International Conference on Robotics and Automation (ICRA).

[9] Thompson N.C., Greenewald K.H., Lee K., Manso G.F. (2020). The Computational Limits of Deep Learning. arXiv.

[10] Sandamirskaya Y. (2022). Rethinking computing hardware for robots. Sci. Robot..

[11] Tavanaei A., Ghodrati M., Reza Kheradpisheh S., Masquelier T., Maida A. (2019). Deep learning in spiking neural networks. Neural Netw..

[12] Schuman C.D., Kulkarni S.R., Parsa M., Mitchell J.P., Date P., Kay B. (2022). Opportunities for neuromorphic computing algorithms and applications. Nat. Comput. Sci..

[13] Kendall J.D., Kumar S. (2020). The building blocks of a brain-inspired computer. Applied Physics Reviews.

[14] Zaidel Y., Shalumov A., Volinski A., Supic L., Ezra Tsur E. (2021). Neuromorphic NEF-Based Inverse Kinematics and PID Control. Front. Neurorobotics.

[15] Volinski A., Zaidel Y., Shalumov A., DeWolf T., Supic L., Ezra-Tsur E. (2022). Data-driven artificial and spiking neural networks for inverse kinematics in neurorobotics. Patterns.

[16] Davies M., Wild A., Orchard G., Sandamirskaya Y., Guerra G.A.F., Joshi P., Plank P., Risbud S.R. (2021). Advancing Neuromorphic Computing With Loihi: A Survey of Results and Outlook. Proc. IEEE.

[17] Modha D.S. (2016). The Brain’s Architecture, Efficiency on a Chip. https://www.ibm.com/blogs/research/2016/12/the-brains-architecture-efficiency-on-a-chip/.

[18] Modha D.S. (2022). Products–Akida Neural Processor SoC. https://brainchip.com/akida-neural-processor-soc/.

[19] Sandamirskaya Y., Kaboli M., Conradt J., Celikel T. (2022). Neuromorphic computing hardware and neural architectures for robotics. Sci. Robot..

[20] Li Y., Ang K.W. (2021). Hardware Implementation of Neuromorphic Computing Using Large-Scale Memristor Crossbar Arrays. Adv. Intell. Syst..

[21] Zhang X., Lu J., Wang Z., Wang R., Wei J., Shi T., Dou C., Wu Z., Zhu J., Shang D. (2021). Hybrid memristor-CMOS neurons for in-situ learning in fully hardware memristive spiking neural networks. Sci. Bull..

[22] Payvand M., Moro F., Nomura K., Dalgaty T., Vianello E., Nishi Y., Indiveri G. (2022). Self-organization of an inhomogeneous memristive hardware for sequence learning. Nat. Commun..

[23] Kimura M., Shibayama Y., Nakashima Y. (2022). Neuromorphic chip integrated with a large-scale integration circuit and amorphous-metal-oxide semiconductor thin-fil msynapse devices. Sci. Rep..

[24] Kim H., Mahmoodi M.R., Nili H., Strukov D.B. (2021). 4K-memristor analog-grade passive crossbar circuit. Nat. Commun..

[25] Gerstner W., Kistler W.M., Naud R., Paninski L. (2014). Neuronal Dynamics.

[26] Bing Z., Meschede C., Röhrbein F., Huang K., Knoll A.C. (2018). A Survey of Robotics Control Based on Learning-Inspired Spiking Neural Networks. Front. Neurorobotics.

[27] Javanshir A., Nguyen T.T., Mahmud M.A.P., Kouzani A.Z. (2022). Advancements in Algorithms and Neuromorphic Hardware for Spiking Neural Networks. Neural Comput..

[28] Guo W., Fouda M.E., Eltawil A.M., Salama K.N. (2021). Neural Coding in Spiking Neural Networks: A Comparative Study for Robust Neuromorphic Systems. Front. Neurosci..

[29] Juarez-Lora A., Ponce-Ponce V.H., Sossa H., Rubio-Espino E. (2022). R-STDP Spiking Neural Network Architecture for Motion Control on a Changing Friction Joint Robotic Arm. Front. Neurorobotics.

[30] Harris C.R., Millman K.J., Van Der Walt S.J., Gommers R., Virtanen P., Cournapeau D., Wieser E., Taylor J., Berg S., Smith N.J. (2020). Array programming with NumPy. Nature.

[31] Meurer A., Smith C.P., Paprocki M., Čertík O., Kirpichev S.B., Rocklin M., Kumar A., Ivanov S., Moore J.K., Singh S. (2017). SymPy: Symbolic computing in Python. PeerJ Comput. Sci..

[32] Eshraghian J.K., Ward M., Neftci E., Wang X., Lenz G., Dwivedi G., Bennamoun M., Jeong D.S., Lu W.D. (2021). Training spiking neural networks using lessons from deep learning. arXiv.

[33] Saito T. (2020). Piecewise linear switched dynamical systems: A review. Nonlinear Theory Its Appl. IEICE.

